# Immunodepletion of high-abundant proteins from acute and chronic wound fluids to elucidate low-abundant regulators in wound healing

**DOI:** 10.1186/1756-0500-3-335

**Published:** 2010-12-14

**Authors:** Lars Steinsträßer, Frank Jacobsen, Tobias Hirsch, Marco Kesting, Caroline Chojnacki, Christoph Krisp, Dirk Wolters

**Affiliations:** 1 Department of Plastic Surgery and Burn Center, BG University Hospital Bergmannsheil, Ruhr-University Bochum, Germany; 2 Department of Analytical Chemistry, Ruhr-University Bochum, Germany

## Abstract

**Background:**

The process of wound healing consists of several well distinguishable and finely tuned phases. For most of these phases specific proteins have been characterized, although the underlying mechanisms of regulation are not yet fully understood. It is an open question as to whether deficits in wound healing can be traced back to chronic illnesses such as diabetes mellitus. Previous research efforts in this field focus largely on a restricted set of marker proteins due to the limitations detection by antibodies imposes. For mechanistic purposes the elucidation of differences in acute and chronic wounds can be addressed by a less restricted proteome study. Mass spectrometric (MS) methods, e.g. multi dimensional protein identification technology (MudPIT), are well suitable for this complex theme of interest. The human wound fluid proteome is extremely complex, as is human plasma. Therefore, high-abundant proteins often mask the mass spectrometric detection of lower-abundant ones, which makes a depletion step of such predominant proteins inevitable.

**Findings:**

In this study a commercially available immunodepletion kit was evaluated for the detection of low-abundant proteins from wound fluids. The dynamic range of the entire workflow was significantly increased to 5-6 orders of magnitude, which makes low-abundant regulatory proteins involved in wound healing accessible for MS detection.

**Conclusion:**

The depletion of abundant proteins is absolutely necessary in order to analyze highly complex protein mixtures such as wound fluids using mass spectrometry. For this the used immunodepletion kit is a first but important step in order to represent the entire dynamic range of highly complex protein mixtures in the future.

## Background

In times of nutritious superabundance obesity becomes a serious concern. Thus, almost 6% of the world's population is suffering from diabetes mellitus [1]. Nearly all types of diabetes mellitus show numerous long-term consequences, such as a heart attack or apoplectic stroke. Diabetics often tend to build ulcers on lower extremities because of arterial obstructive diseases. These wounds show an abnormal wound healing process, but the reason for this dysfunction is still unknown today. Several important components like growth factors or cytokines etc. were characterized recently, which enable a classification of the wound healing process in different phases [2,3]. Nevertheless, the underlying mechanisms in wound healing disturbance have not yet been answered completely. Consequently, understanding of the factors involved and their interactions, particularly on the biomolecular level, is of great interest.

Some common biological and chemical methods, such as ELISA or 2 D gel electrophoresis, are available for the analysis of protein mixtures nowadays, but they require either an accurate knowledge of the target proteins or they offer a limited dynamic range.

Mass spectrometry (MS) evolved as a powerful tool for the analysis of complex protein mixtures. Multidimensional protein identification technology (MudPIT) for shotgun proteomics is one of the latest accomplishments in proteome analysis [4,5].

MudPIT is a gel-free approach and combines two-dimensional liquid chromatography with electrospray-ionization (ESI)-MS/MS, which comprises a relatively large dynamic range of 4 orders of magnitude. Several hundred proteins can be generated within 24 h employing this technology.

We utilized MudPIT for the proteomic analysis of acute and diabetic wound fluid to achieve a deeper insight into wound healing processes in the future. Due to the large nescience of the proteins involved and their abundance in wound fluids, the work with exudates is based on the knowledge already gained from human plasma. More than 90% of the human plasma proteome consists of highly abundant proteins, which obscure the identification results [6,7]. It has been reported that these abundant proteins are also present in wound fluid and that their depletion is necessary for the identification of proteins present in lower concentrations, which are supposed to have a significant influence in disturbed wound healing [8]. Nevertheless, the dynamic range of a consecutive depletion and mass spectrometric approach was not investigated and low-abundant proteins have not been detected. Therefore, the goal of this study was to evaluate the biological handling of wound fluids for the depletion of the 20 most-abundant proteins, and the mass spectrometric detection of low-abundant regulatory proteins involved in wound healing.

## Methods

### Collection of Wound Fluid Samples

As part of the inpatient treatment of acute and chronic wound polyvenylalcohol (PVA) sponges were applied to the wound bed to withdraw the exudates. Soaked sponges were then moistened with 2 ml protease inhibitor using Roche Complete tablets containing a protease inhibitor cocktail of serine, cysteine and metalloprotease inhibitors (Penzberg, Germany) dissolved in saline and carefully centrifuged at 125 × *g *to collect the wound fluid. This step was repeated twice. One tablet was dissolved in 50 ml saline solution. Cell debris was removed by centrifugation at 14.000 × *g *for 5 min from the supernatant, which was snap-frozen with liquid N_2 _and stored at -80°C until further analysis. Samples were collected from three patients showing acute wounds and from three chronic wound patients with diabetes mellitus type 2. The protein content of the exudates was determined to be 20-50 mg/ml by bicinchoninic acid (BCA) assay (BCA Protein Assay Kit, Pierce Chemicals, Rockford, IL) and samples were diluted to yield a final protein content of 15 mg. The ethics committee of the Ruhr-University Bochum, Germany approved the study protocol.

### Immunodepletion

#### Depletion of the 20 most abundant proteins in acute and chronic wound fluids

In order to become familiarized with depletion conditions and working routines for wound fluid we tested the depletion efficiency of the ProteoPrep^® ^20 Plasma Immunodepletion Kit (Sigma-Aldrich, Steinheim, Germany). The ProteoPrep^® ^20-Kit depletes the 20 most abundant proteins in human plasma, listed in Table 1.

**Table 1:** Twenty most-abundant proteins depleted by ProteoPrep® 20

Albumin	α1-Acid-Glycoprotein
IgG	Ceruloplasmin

IgA	Apolipoprotein A-I

IgM	Apolipoprotein A-II

IgD	Apolipoprotein B

Transferrin	Complement C1q

Fibrinogen	Complement C3

α2-Macroglobulin	Complement C4

α1-Antitrypsin	Plasminogen

Haptoglobin	Prealbumin

These proteins, which occur in acute and chronic wound fluid, were removed with this kit according to the manufacturer's instructions.

The ProteoPrep^® ^20 device handles volumes of about 8 µl of serum (diluted to about 100 µl working volume) per run. We decided to use 1 ml of wound fluid (15 mg protein content) in 10 consecutive runs, combine and concentrate the depleted fractions and proceed with a 11th final depletion step to achieve 99% depletion as suggested by the manufacturer. The flow-through proteins, i.e. the low concentrated depleted ones, were acetone precipitated and resuspended in 25 mM NH_4_HCO_3_. The bound proteins, i.e. the depleted 20 most abundant ones, were TCA (Trichloroacetic acid) precipitated and resuspended in 25 mM NH_4_HCO_3 _as well. 1D-SDS-PAGE and MudPIT analyzed the resulting depleted concentrated fractions.

#### Gel electrophoresis

Wound fluid samples (20 µg each) were incubated for 10 min at 60°C in presence of 2 times sample buffer (0.5 M Tris-HCL, pH 6.8, 3% w/v SDS, 10% v/v glycerol,0.5% v/v 2-mercaptoethanol, and bromophenol blue). Samples were loaded onto a 15% sodium dodecyl sulfate-polyacrylamide gel (SDS-PAGE) and run at a constant voltage of approximately 100 V. Silver staining was applied to visualize proteins.

#### Measurement of IL6 concentration

The level of Interleukin-6 concentrations in crude wound exudates and the corresponding depleted flow through was measured by a specific sandwich enzyme (horseradish peroxidase, HRP)-linked immunosorbent assay (ELISA) (R&D, Wiesbaden, Germany) following the standard protocol provided. The concentrations were calculated using a standard curve that was created by serially diluted IL-6 standard protein (R&D, Wiesbaden, Germany) by the Elx808 Microplate Reader (BioTek, Bad Friedrichshall, Germany) and Excel software (Microsoft, Unterschleißheim, Germany).

#### MudPIT

A quaternary Ultimate High performance liquid chromatography (HPLC)-pump (Dionex, Amsterdam, Netherlands) was connected to a Thermo LTQ XL ion trap mass spectrometer from Thermo Fisher Scientific Inc. (San Jose, CA).

600 µg of each depleted sample were digested enzymatically with the proteases trypsin (sequencing grade, Promega, Madison, WI) and chymotrypsin (modified sequencing grade, Princeton Separations Inc., Adelphia, NJ).

The peptides generated were loaded onto a biphasic micro capillary (diameter (d) = 100 µm) which was packed with 10 cm of Luna C_18 _reversed phase (RP) Material, 5 cm strong cation exchange (SCX) material (Phenomenex, Germany) and finally with 6 cm of Luna C_18 _RP material. The effective flow rate of the column was set to 150 - 250 nL/min and a spray voltage of 1.8 kV was supplied. The LTQ XL was operated via instrument method files in the Sequence Setup window of Xcalibur. The heated desolvation capillary was set to 180 °C. Chromatography was performed as described elsewhere [5], using a 20 step gradient program including salt steps with 5, 10, 15, 20, 25, 30-100% 250 mM Ammonium acetate (NH_4_Ac) and 33, 50, 75% 1.5 M NH_4_Ac.

A full MS scan between 400 and 2000 m/z for the precursor ion was applied followed by full MS/MS scans of the three most intense ions from the preceding MS scan.

### Data Analysis

To interpret the MS/MS data we used the SEQUEST algorithm implemented in Bioworks 3.3.1. Data were searched against the human Swiss-Prot database release 54. All accepted results had a ∆Cn value of at least 0.08 and singly, doubly and triply charged peptides had to be partially tryptic or chymotryptic with XCorr > 1.8, 2.5 or 3.5 and a precursor mass accuracy of at least 2.5 atomic mass units (amu). Fragment ion tolerance was set to 1 amu. Methionine oxidation was considered a variable modification. The rank of the preliminary score (RSp) was set ≤ 10.

A protein was considered as present in the mixture when two or more unique peptides were identified by the previous criteria. The lists of the identified proteins were generated with DTAselect 1.9. False positive rate was less than 2% estimated by a reverse database search. The false discovery rate was calculated by dividing the absolute number of hits from this reversed database search by the sum of hits from both database searches (reversed database and actual database).

The depletion efficiency of high abundant proteins in wound fluids was estimated comparing the spectrum count percentages of these proteins in the enriched sample, called bound fraction, and the depleted sample, in the following named as flow-through fraction. Spectrum count is given as the number of successful MS/MS experiments. Percentages were calculated dividing the spectrum count of each protein by a hundredth of the total spectrum count of each experiment.

## Results

### Depletion of high-abundant proteins in acute and chronic wound fluids

**Figure 1:**
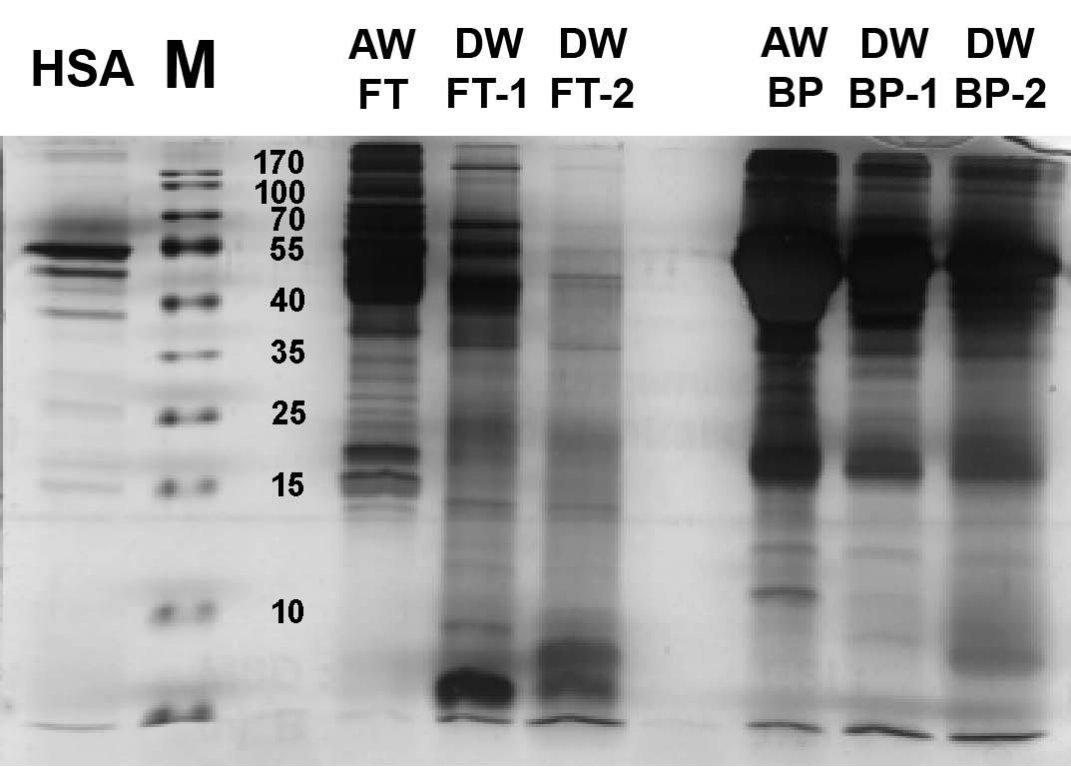
**1D-SDS-PAGE (silver stained)**. Bound and flow-through (FT) proteins derived from diabetic and acute wound fluids. Lane 1: Human serum albumin (HSA) (2 µg), 2: MW marker, 3: empty, 4: FT proteins acute, 5-6: FT proteins diabetic (two different patients), 7: empty, 8-10: bound proteins (8: acute; 9-10: diabetic).

We monitored the degree of depletion of the 20 most abundant proteins in the bound fraction and the flow-through fraction, by using 1D-SDS PAGE and MudPIT. The bound fraction should contain the majority of those 20 proteins, whereas the flow-through supposedly contains only the low abundant proteins and only trace amounts of the 20 high abundant proteins. It seems very likely and the results from Fernandez *et al*. indicate that the 20 high-abundant proteins are also present in wound fluids [8]. However, it was never shown that their amount and distribution in human wound fluids are suitable for the ProteoPrep^® ^20 Kit, which consequently yields identification of low-abundant proteins. 1D-SDS-PAGE analysis of the bound fraction exhibits a successful depletion of the high-abundant proteins in the mass range greater than 25 kDa, when using the ProteoPrep^® ^20 Kit (Figure 1). Lanes 8-10 reflect the bound fraction from acute and diabetic wound exudates, respectively. Spot pattern of the high-abundant proteins in lanes 8-10 are nearly identical, therefore we suppose, that the 20 most abundant proteins have a predominant occurrence in human wound fluid similar to human plasma (data not shown). For further evaluation of this aspect we performed a MudPIT analysis of the bound protein fraction from a diabetic wound fluid sample (Table 2).

**Table 2:** Detection of the 20 most-abundant proteins of the bound fraction.

Protein code	Spectral count	Sequence count	Sequence coverage [%]
*P02768|ALBU_HUMAN*	2119 (51.68)	87	43

*P01009|A1AT_HUMAN*	307 (7.49)	29	36

*P02763|A1AG1_HUMAN*	230 (5.61)	18	34

*P19652|A1AG2_HUMAN*	31 (0.76)	10	27

*P01857|IGHG1_HUMAN*	120 (2.93)	26	32

*P01859|IGHG2_HUMAN*	118 (2.88)	22	28

*P01860|IGHG3_HUMAN*	45 (1.10)	10	17

*P02647|APOA1_HUMAN*	25 (0.61)	10	23

P02652|APOA2_HUMAN	1 (0.02)	1	9

*P04114|APOB_HUMAN*	100 (2.44)	28	6

*P01876|IGHA1_HUMAN*	19 (0.46)	6	19

*P01877|IGHA2_HUMAN*	15 (0.37)	6	20

*P02671|FIBA_HUMAN*	25 (0.61)	7	6

*P02675|FIBB_HUMAN*	26 (0.63)	12	19

*P02679|FIBG_HUMAN*	25 (0.61)	12	14

*P02787|TRFE_HUMAN*	61 (1.49)	11	7

*P01023|A2MG_HUMAN*	47 (1.15)	25	17

P01871|MUC_HUMAN	3 (0.07)	2	6

*P00738|HPT_HUMAN*	55 (1.34)	14	17

*P00450|CERU_HUMAN*	114 (2.78)	26	14

P02766|TTHY_HUMAN	322 (7.85)	9	24

P00747|PLMN_HUMAN	2 (0.05)	1	1

*P01024|CO3_HUMAN*	268 (6.54)	52	18

***Additionally detected***			

*P01834|KAC_HUMAN*	17 (0.41)	2	16

*P01031||CO5_HUMAN*	3 (0.07)	3	2

*P09871|C1S_HUMAN*	2 (0.05)	2	4

***Not detected***			

*P02746|C1QB_HUMAN*	-	-	-

*P02747|C1QC_HUMAN*	-	-	-

*P01880|IGHD_HUMAN*	-	-	-

*P01880|CO4_HUMAN*	-	-	-

This table shows the identification rate of the 20 most-abundant proteins that bound to the kit's resin after depletion with the ProteoPrep^® ^20 Plasma Immunodepletion Kit. Percentage of the spectral count is given in brackets

Proteins in the flow-through, lanes 4-6, revealed differences in spot pattern compared to the bound fraction, especially in the intensity of spots in higher mass ranges. Based on these qualitative results, we assume that the depletion of the 20 most abundant proteins was successful. We validated this assumption by MudPIT analyses.

### MudPIT analyses

#### High-abundant proteins

The MudPIT analysis of the bound protein fraction revealed 17 of the 20 most abundant proteins, enriched by the kit resin (Table 2). Overall, more than 20 entries are shown in Table 2, contributed to polyclonal antibodies in the ProteoPrep^® ^20 Kit, which do not discriminate different isotype subclasses, such as IGHG1_Human, IGHG2_Human or IGHG3_Human. 14 of these proteins were found with more than one peptide in the bound fraction. Due to quantitative evaluation of the bound fraction, the two peptides per protein criteria were not applied for the high-abundant proteins. Thus, three proteins from the depletion list of the kit were identified with only one peptide per protein in this sample.

Furthermore, we were able to deplete three additional high-abundant proteins, two from the complement system (CO5_HUMAN, C1S_HUMAN) and one immunoglobulin (KAC_HUMAN), due to known protein-protein interactions or cross reactivity. Merely IgD, complement C4 and complement C1q could not be detected by MudPIT analysis. However, it is possible that these proteins are either not expressed in human wound fluid or down-regulation in the present state avoids their detection. Furthermore these proteins are in the lower concentration range of the 20 most abundant proteins, therefore masking of these proteins might be possible, or irreversible binding on the kit resin could be a reason for these results. These results show that 17 of the most abundant plasma proteins also occur in human wound fluids.

In total, 78 proteins (sequence count > 1) were identified in the bound fraction of the MudPIT experiment. The abundant proteins retained the additional 61 proteins. However, this effect is most likely due to protein-protein interaction and unspecific binding, which does not significantly influence the identification rate in the MudPIT experiments of the depleted fraction. Almost 70% of these additional proteins were identified in the flow-through fraction as well. According to the low number of spectral counts from these unspecifically bound proteins in the bound fraction, the analysis of the flow-through fraction is not largely affected. Spectrum count is defined as the number of successful MS/MS spectra. It is related to the protein amount in the sample and proteins can be quantified relative to one other [9].

#### Low abundant proteins

We tested the ProteoPrep^® ^20 Plasma Immunodepletion Kit with three chronic and three acute wound fluid samples. To demonstrate a successful depletion of these 20 high-abundant plasma proteins in wound fluids, we applied MudPIT analyses to the flow-through fractions of the acute and chronic wound fluid samples. After MudPIT experiments we monitored the amount of high-abundant proteins in the flow-through and quantified them by spectral counting.

We focused on the degree of depletion. In all samples, the majority of the high-abundant proteins identified exhibited relatively low spectral counts compared to the total number of spectral counts obtained for such samples. Usually, the spectral count percentage of the 20 high-abundant proteins is below 1% for each protein (Table 3). Some proteins showed spectral counting percentages above 1% such as albumin and apolipoprotein A1. Six of the 20 most abundant proteins were not identified in the depleted fractions, neither in the chronic samples nor in the acute ones.

**Table 3:** Evaluation of the 20 most abundant proteins in the flow-through fractions.

Protein code	DWF 1	DWF 2	DWF3	AWF 1	AWF 2	AWF3
**Spectral count total**	**12386**	**6559**	**9027**	**8995**	**6318**	**7819**

*P02768|ALBU_HUMAN*	126 (1.02)	7 (0.11)	119 (1.32)	4 (0.07)	119 (1.88)	33 (0.42)

*P01009|A1AT_HUMAN*	3 (0.07)	-	-	1 (0.01)	7 (0.11)	196 (2.51)

*P02763|A1AG1_HUMAN*	27 (0.21)	-	-	61 (0.68)	-	71 (0.91)

*P19652|A1AG2_HUMAN*	1 (0.01)	-	-	-	-	55 (0.70)

*P01857|IGHG1_HUMAN*	30 (0.24)	-	40 (0.44)	-	37 (0.59)	-

*P01859|IGHG2_HUMAN*	23 (0.19)	6 (0.09)	29 (0.32)	2 (0.02)	34 (0.54)	16 (0.20)

*P01860|IGHG3_HUMAN*	15 (0.12)	5 (0.08)	4 (0.04)	-	14 (0.22)	4 (0.05)

*P02647|APOA1_HUMAN*	17 (0.14)	1 (0.02)	9 (0.10)	-	30 (0.48)	142 (1.82)

P02652*|*APOA2_HUMAN	1 (0.01)	-	-	-	1 (0.02)	1 (0.01)

*P04114|APOB_HUMAN*	21 (0.17)	1 (0.02)	18 (0.20)	2 (0.02)	43 (0.68)	32 (0.41)

*P01876|IGHA1_HUMAN*	-	-	6 (0.07)	-	-	-

*P01877|IGHA2_HUMAN*	-	-	-	-	-	-

*P02671|FIBA_HUMAN*	58 (0.47)	50 (0.76)	33 (0.37)	2 (0.02)	50 (0.79)	13 (0.17)

*P02675|FIBB_HUMAN*	12 (0.10)	1 (0.02)	17 (0.19)	3 (0.03)	4 (0.06)	-

*P02679|FIBG_HUMAN*	2 (0.05)	5 (0.08)	6 (0.07)	1 (0.01)	3 (0.05)	-

*P02787|TRFE_HUMAN*	1 (0.01)	-	8 (0.09)	-	10 (0.16)	1 (0.01)

*P01023|A2MG_HUMAN*	11 (0.09)	5 (0.08)	50 (0.55)	3 (0.03)	26 (0.41)	4 (0.05)

P01871|MUC_HUMAN	-	-	-	-	-	-

*P00738|HPT_HUMAN*	60 (0.49)	8 (0.12)	5 (0.06)	2 (0.02)	22 (0.35)	52 (0.67)

*P00450|CERU_HUMAN*	3 (0.02)	-	1 (0.01)	-	-	-

P02766|TTHY_HUMAN	-	-	71 (0.79)	-	164 (2.60)	16 (0.2)

P00747|PLMN_HUMAN	-	-	1 (0.01)	-	-	-

*P01024|CO3_HUMAN*	44 (0.36)	10 (0.15)	68 (0.75)	-	30 (0.47)	3 (0.04)

*P01834|KAC_HUMAN*	4 (0.03)	11 (0.17)	47 (0.52)	1 (0.01)	23 (0.36)	-

*P01031||CO5_HUMAN*	4 (0.03)	1 (0.02)	20 (0.22)	2 (0.02)	14 (0.22)	19 (0.24)

*P09871|C1S_HUMAN*	1 (0.01)	-	10 (0.11)	-	6 (0.09)	1 (0.01)

*P02746|C1QB_HUMAN*	-	-	-	-	-	-

*P02747|C1QC_HUMAN*	-	-	-	-	-	-

*P01880|IGHD_HUMAN*	-	-	-	-	-	-

*P01880|CO4_HUMAN*	-	-	-	-	-	-

Three diabetic (DWF) and three acute wound fluids (AWF) were analyzed after depletion with the ProteoPrep^® ^20 Plasma Immunodepletion Kit. Spectral counts were standardized in respect to the overall number and percentages are given in brackets.

**Figure 2:**
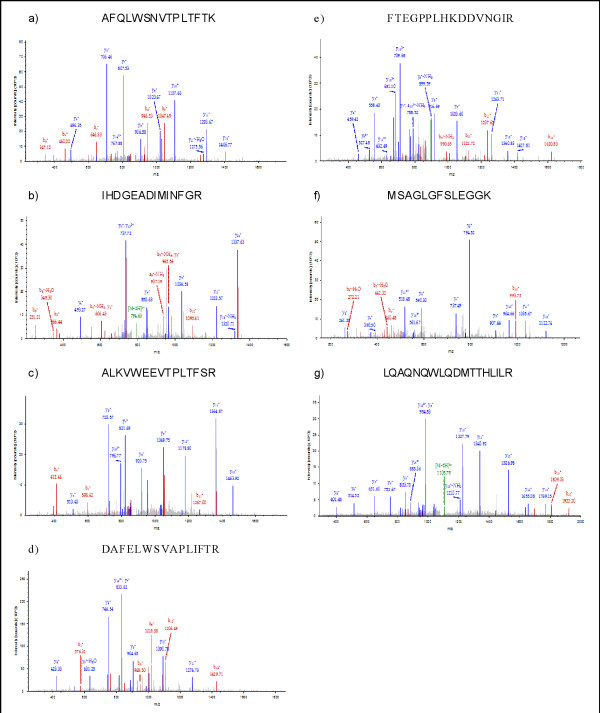
**MS/MS spectra of a) MMP1 (Peptide mass: 876 m/z), XCorr 5.19 b) MMP2 (794 m/z), XCorr: 4.30 c) MMP3 (888 m/z), XCorr: 4.07 d) MMP8 (926 m/z), XCorr: 5.11 e) MMP9 (879 m/z), XCorr: 5.01 f) IL16 (627 m/z), XCorr: 3.90 and g) IL6 (1105 m/z), XCorr 4.11; all peptides are doubly charged**.

Overall, 355 unique proteins (data not shown) were identified by the MudPIT experiments in the depleted fractions. Since this work is technology driven, it is beyond the scope of this work to analyze these proteins in detail. However, to demonstrate the opportunity of this depletion strategy, we briefly analyzed the list of identified proteins, which are usually considered as low abundant. Anderson and Anderson demonstrated the difficulties in mass spectrometric detection of very low concentrated human plasma proteins [6]. For example interleukins and interferons exist in pg/ml concentrations in human plasma and hence are considered as low abundant. With the depletion strategy we were able to detect low-abundant proteins such as Interleukin-16 (Q14005|IL16_HUMAN) and Interleukin 6 (P05231|IL6_HUMAN) (Figure 2). Interleukins play a very important role in wound healing mechanisms, especially Interleukin-16 and Interleukin-6, which are supposed to have proinflammatory effects [10,11]. Furthermore, matrix metalloproteinases (MMP) 1, 2, 3, 8 and 9 could be identified. Exemplified spectra of the respective peptides are shown in Figure 2. MMPs are mainly involved in the degradation of extracellular matrix proteins. In human plasma the amount varies in the pg/ml range, thus they are considered as low abundant. MMPs are largely described as up regulated in several types of wound tissue, or fluids and therefore discussed as potential biomarker [12-16].

Since Anderson and Anderson determined the proteome profile from human plasma, its values might not be applicable to human wound fluid at all. Therefore, an ELISA assay for IL-6 was established. An IL-6 concentration of 29 ng/ml was measured within the depleted wound fluid sample, while it was below background level within the captured high-abundant protein fraction.

At the high-abundance end the 22 most abundant proteins comprise more than 99% of all proteins in human plasma, whereas human serum albumin itself comprises 65% [6,17]. We assume that analogous to plasma high-abundant proteins were removed by the kit. 15 mg protein per undepleted wound fluid sample was applied to the kit resin. After depletion the samples yield a protein concentration of at most 1 mg in the flow-through fraction, according to the BCA assay. BCA assays, performed before and after depletion with the ProteoPrep^® ^kit, indicated a reduction of the total protein amount of almost 93%. Consequently, effective depletion of high abundant proteins facilitates the identification of low abundant proteins.

## Discussion

Diabetic patients often tend to build ulcers on their lower extremities, whereas the pathophysiology of the impaired wound healing in these patients remains unclear. However, there are many expensive treatment options (e.g. dressings, growth factors) for these wounds available in clinics. Unfortunately, the importance for medical device companies to demonstrate a clinical benefit for patients on the molecular level is small. Therefore, central questions about impaired wound healing, especially the biomolecular aspects remain open. Knowledge of the human wound fluid proteome might help to address some of the questions, since new methods and technology such as new developments in mass spectrometry are constantly emerging.

Human wound fluid comprises similar to human plasma a very complex proteome, but very few proteins are investigated in detail until now [3,12]. Since large-scale analyses have not yet elucidated the wound fluid proteome, the human plasma proteome is the next best choice to correlate information about protein composition or function. It is feasible that abundant proteins in plasma play a substantial and comparable role in human wound fluid, too. Nevertheless, it has not been proven in detail that immunodepletion strategies applied to human serum or plasma are comparably efficient for human wound fluid.

High-abundant protein species have a huge impact on the dynamic range in mass spectrometric analysis by masking low-abundant proteins and thereby reduce the identification potential. This makes depletion steps of high-concentrated proteins essential. The ProteoPrep^® ^20 Plasma Immunodepletion Kit (Sigma-Aldrich, Steinheim, Germany) depletes the 20 most-abundant proteins in human plasma. We have applied this kit for the use on wound fluids and showed qualitatively by 1D-SDS-PAGE and quantitatively by MudPIT experiments that depletion was successful. MudPIT analysis of the bound fraction revealed 17 of the 20 most-abundant proteins from human plasma to be present in wound fluid. It can be assumed that these proteins are not responsible for wound healing dysfunction, therefore techniques have to be developed, which make lower abundant proteins accessible. These proteins are usually addressed by ELISA, e.g. for the ratio estimation of MMPs and their corresponding endogenous inhibitors, the tissue inhibitors of metalloproteinases (TIMPs). This ratio has to be in balance for an undisturbed wound healing process. Investigations showed that decreased MMP3 and MMP9 levels during therapy correlate with positive wound healing outcomes [18]. These facts are just an indication for patients' treatment and relevant investigation methods, but they have to be expanded upon to elucidate the chronic wound problem.

For mass spectrometric analysis of low abundant proteins in wound fluids, depletion strategies for high abundant proteins are necessary. We tested the ProteoPrep^® ^Plasma 20 immunodepletion kit for the use of protein depletion in chronic and acute wound fluids. The quality of depletion using the immunodepletion kit was assessed by comparing spectrum count percentages of high abundant proteins in the enriched and the flow-through fractions, collected separately. SDS-PAGE separations (Figure 1) and BCA assay measurements indicate a substantial decrease in the amount of proteins in the depleted samples. MudPIT experiments prove that lower protein content is related to the depletion of the most abundant proteins. The bound fraction contained 17 of the 20 most abundant proteins, while three were not detectable. In the flow-through fractions of three acute and three diabetic samples, six high-abundant proteins could not be identified e.g. complement system proteins and immunoglobulins. The majority of the detected abundant proteins in the depleted samples show a spectrum count percentage of less than 1% each, compared to the overall count. These concentrations of the 20 most-abundant proteins in the depleted fraction are for future investigations tolerable. Due to the successful depletion, the accessible dynamic range should be expanded. So far mass spectrometric approaches including 2D-PAGE or MudPIT comprise a dynamic range of 10^2^-10^4 ^[5,6]. In this study we determined a dynamic range of at least 5 to 6 orders of magnitude, since we identified low-abundant proteins such as MMP1, MMP2, MMP3, MMP8, MMP9, IL6 and IL16 (Figure 2). Interestingly, Anderson and Anderson, and Kilpadi et al. presented concentrations for these proteins in human plasma or swine serum respectively in the order of pg/ml [6,19], whereas our data showed significantly higher values for IL6 with 20-40 ng/ml in wound fluids. Similar observations were obtained by Kilpadi et al. who investigated wound fluids from patients treated with the vacuum assisted closure therapy [18]. They reported concentrations between 151-666 ng/ml for MMP3 and 1,593-5,649 ng/ml for MMP9 in these fluids. The results lead to the assumption that the protein pool in human wound fluids differs at least in respect to protein concentration compared to human plasma. The removal of high-abundant proteins is crucial for a detailed analysis of low-abundant proteins in order to create a comprehensive protein profile of chronic and acute wound fluids in the future. This facilitates a deeper insight in the physiology and pathophysiology of wound repair which will hopefully contribute towards establishing new therapy strategies.

## Competing interests

The authors declare that they have no competing interests.

## Authors' contributions

CC, FJ, TH, MK and DW have performed most of the experiments. LS, FJ and DW participated in experimental design, data interpretation and writing of the manuscript.

All authors read and approved the final version of this manuscript.
